# Association between erythrocyte dynamics and vessel remodelling in developmental vascular networks

**DOI:** 10.1098/rsif.2021.0113

**Published:** 2021-06-23

**Authors:** Qi Zhou, Tijana Perovic, Ines Fechner, Lowell T. Edgar, Peter R. Hoskins, Holger Gerhardt, Timm Krüger, Miguel O. Bernabeu

**Affiliations:** ^1^ School of Engineering, Institute for Multiscale Thermofluids, The University of Edinburgh, Edinburgh, UK; ^2^ Centre for Medical Informatics, Usher Institute, The University of Edinburgh, Edinburgh, UK; ^3^ The Bayes Centre, The University of Edinburgh, Edinburgh, UK; ^4^ Centre for Cardiovascular Science, The University of Edinburgh, Edinburgh, UK; ^5^ Max-Delbrück-Center for Molecular Medicine in the Helmholtz Association (MDC), Berlin, Germany; ^6^ Biomedical Engineering, University of Dundee, Dundee, UK; ^7^ Vascular Patterning Laboratory, Department of Oncology and Leuven Cancer Institute (LKI), KU Leuven, Belgium; ^8^ DZHK (German Center for Cardiovascular Research), Germany; ^9^ Berlin Institute of Health, Germany

**Keywords:** angiogenesis, haemodynamics, microcirculation, vascular remodelling, wall shear stress, red blood cells

## Abstract

Sprouting angiogenesis is an essential vascularization mechanism consisting of sprouting and remodelling. The remodelling phase is driven by rearrangements of endothelial cells (ECs) within the post-sprouting vascular plexus. Prior work has uncovered how ECs polarize and migrate in response to flow-induced wall shear stress (WSS). However, the question of how the presence of erythrocytes (widely known as red blood cells (RBCs)) and their impact on haemodynamics affect vascular remodelling remains unanswered. Here, we devise a computational framework to model cellular blood flow in developmental mouse retina. We demonstrate a previously unreported highly heterogeneous distribution of RBCs in primitive vasculature. Furthermore, we report a strong association between vessel regression and RBC hypoperfusion, and identify plasma skimming as the driving mechanism. Live imaging in a developmental zebrafish model confirms this association. Taken together, our results indicate that RBC dynamics are fundamental to establishing the regional WSS differences driving vascular remodelling via their ability to modulate effective viscosity.

## 1. Introduction

Sprouting angiogenesis is an essential vascularization mechanism and consists of two well-differentiated phases: sprouting and remodelling [1,2]. During the sprouting phase, a primitive network of vessels is laid out in response to proangiogenic factors via a well-established programme of cellular and molecular events [3]. The remodelling phase is responsible for overhauling this primitive network into a hierarchical structure that can efficiently implement the transport capabilities of the cardiovascular system. During the remodelling phase, extensive vessel pruning is achieved primarily via dynamic rearrangement of endothelial cells (ECs) [4].

The mechanobiological regulation of ECs has been extensively studied, and it is known that ECs respond to their haemodynamic environment [5]. Studies in various animal models have shown that blood flow provides mechanical cues to drive vascular remodelling (e.g. chick embryo [6], mouse yolk sac [7,8], mouse aortic arch [9], zebrafish embryo [4,10–12] and mouse retina [4]). Furthermore, these studies have uncovered multiple molecular regulators of EC response to blood shear stress, such as VEGF [13], Wnt [14], Notch [15,16], TGF*β*/BMP [17–19], DACH1 [20] and KLF2 [21,22].

In previous works, we demonstrated that blood shear stress coordinates EC migratory behaviour to achieve vessel regression during the remodelling phase [4,14,18]. In particular, differences in wall shear stress (WSS) between neighbouring vessel segments lead to polarization and migration of ECs away from vessel segments experiencing low WSS. In these studies, WSS was calculated using a mathematical flow model that assumes blood as a single-phase non-Newtonian fluid (i.e. homogeneous fluid of variable viscosity) without considering the presence of individual red blood cells (RBCs). However, recent computational studies in microscale vessels have demonstrated that RBCs leave transient WSS luminal footprints and therefore could non-trivially modify the local WSS differences driving vascular remodelling [23–25]. This effect of RBCs is closely related to their crucial role in the formation of cell-free layer and the regulation of effective viscosity as well as flow field in microvascular blood flow [26–32].

In the current study, we propose that the cellular nature of blood (i.e. primarily a suspension of RBCs) plays a key role in establishing the local WSS differences driving vascular remodelling. We approach the problem computationally based on the mouse retina model of angiogenesis and provide experimental validation in a developmental zebrafish model. We present simulations of cellular blood flow in vascular networks undergoing remodelling and characterize the bulk flow and RBC dynamics in them. Remarkably, we uncover a previously unreported high-level heterogeneity in RBC perfusion throughout the developing network and a strong association between RBC hypoperfusion and vessel regression, which we further confirm experimentally in our zebrafish model via live imaging. Furthermore, our experiments confirm previous findings with additional insights that the presence of RBCs is required for effective vascular remodelling at a whole plexus level [7]. Finally, we demonstrate that RBC hypoperfusion is primarily driven by the plasma skimming effect, i.e. the uneven distribution of RBC volume fraction at microvascular bifurcations [33,34], but uncover important deviations from existing theory caused by asymmetry of the cross-sectional distribution of haematocrit in some feeding vessels.

In line with our previous findings on WSS-modulated EC migration, we argue here that RBC hypoperfusion constitutes a mechanism for the enhancement of local WSS differences driving vascular remodelling. This is attributed to the direct relationship between RBC volume fraction, effective viscosity and WSS. Additionally, we speculate that vascular remodelling driven by the principle of removing RBC-poor vessels from the primitive vasculature will lead to a network layout that avoids portions of the tissue being vascularized but with poorly oxygenated blood. This RBC-driven process, highly dynamical and emergent in nature, can importantly contribute to the optimal patterning of vascular networks during development. Conversely, it provides a vascular remodelling mechanism to support recent clinical findings in diabetes mellitus and hypertension [35–38] reporting that the onset and progression of microangiopathy may arise from altered RBC mechanical properties (e.g. impaired deformability) that disrupts the microcirculatory blood flow: a hypothesis first proposed by Simpson in the 1980s [39].

## 2. Results

### 2.1. Simulation of cellular blood flow in microvascular network: validation versus *in vivo* data

**Figure 1.**
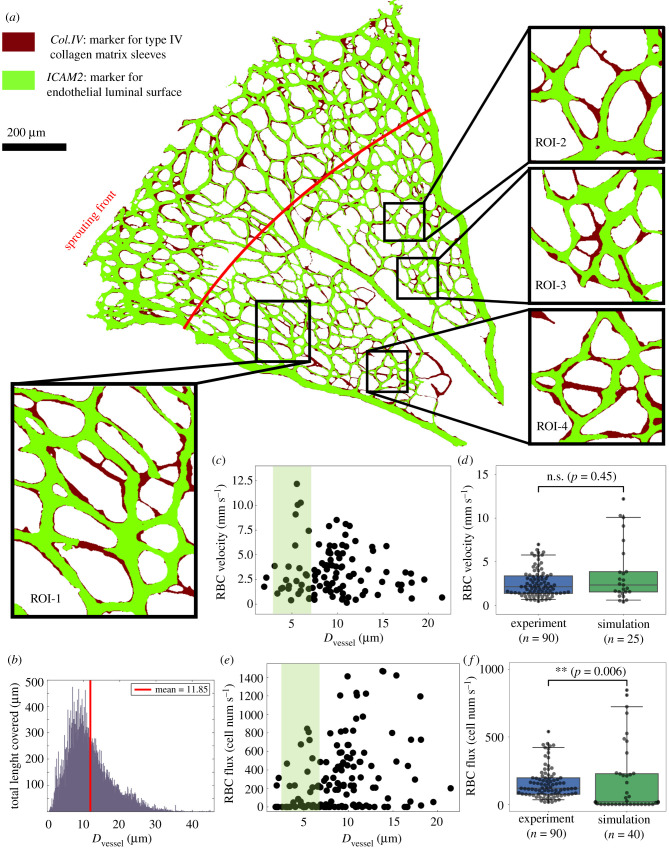
Simulated RBC velocity and cell flux in the primitive vasculature of developing mouse retina. (*a*) A vascular plexus of postnatal day 5 (P5) mouse retina, with vessel lumen and collagen matrix sleeves labelled by ICAM2 (light green) and Col.IV (dark red), respectively. The insets show four regions of interest (ROI-1, ROI-2, ROI-3 and ROI-4) selected from the remodelling region of the plexus. The red line indicates the transitional border between the sprouting and remodelling regions as estimated by [40]. (*b*) Network diameter histogram showing the total length covered by vessel segments of given diameters. (*c*) Simulated RBC velocities and (*e*) RBC fluxes measured in vessel segments from all ROIs. The shaded areas in (*c*,*e*) highlight data from capillary vessels within diameter between 3 and 7 μm. (*d*,*f*) Comparison of simulated RBC velocities and fluxes against recent *in vivo* measurements by [41,42] from *adult* mouse retinal capillaries with the same diameter range (two-sided Mann–Whitney *U* test).

The vascular plexus of a wild-type mouse retina at postnatal day 5 (P5) was imaged and binarized following staining for collagen IV matrix sleeves (Col.IV) and ICAM2 luminal reporter (see protocol in §5.1.1). The ICAM2 mask delineates the perfused vessels in the network while the pixel-wise difference between the Col.IV and ICAM2 masks highlights vessel segments undergoing remodelling (figure 1*a*) as demonstrated in Franco *et al.* [4]. Therefore, the Col.IV mask constitutes a good approximation of the network morphology prior to these remodelling events. Indeed, the network recapitulates the lognormal distribution of vessel diameters previously reported by Bernabeu *et al.* [43], with a maximum value of 45 µm and a mean of 11.85 µm (figure 1*b*). Based on our previously proposed approach [44], we construct a three-dimensional (3D) flow model from the Col.IV binary image and run whole-plexus blood flow simulations under the assumption of generalized non-Newtonian blood rheology (see the methods in §5.2.2). This is followed by simulations of RBC flow in selected regions of interest (ROIs; see the selection criteria in §5.2.1) from the capillary bed (e.g. ROI-1, ROI-2, ROI-3 and ROI-4 in insets of figure 1*a*) with inflow/outflow boundary conditions specified from the whole plexus model (see the methods in §5.2.3). For full details of the computational framework including model configuration and simulation parameters, refer to figures S1–S2 and tables S1–S3 in §S1 of the electronic supplementary material.

Our simulation recapitulates flow rates in the main artery and veins (running in the axial direction of the retina) of 0.36 µl min^−1^ and 0.17–0.19 µl min^−1^, respectively. These flow rates are in good agreement with *in vivo* measurements recently performed in adult mouse retina of 0.39–0.59 µl min^−1^ and 0.24 µL min^−1^, respectively [41]. The RBC velocities and fluxes calculated in the simulated ROIs are in the range 0–12.5 mm s^−1^ (figure 1*c*) and 0–1500 cells s^−1^ (figure 1*e*), respectively. These results are also validated against reported *in vivo* single-cell velocimetry data available for capillaries within diameter 3–7 µm [41,42]. Both the experimental and simulation RBC velocities follow a skewed distribution and show good agreement with median values of 2.14 mm s^−1^ and 2.35 mm s^−1^, respectively (*p* > 0.05, figure 1*d*). The RBC fluxes are significantly higher in experiments than in simulations with median values of 120 cells s^−1^ and 19.3 cells s^−1^, respectively (*p* < 0.01, figure 1*f*). The main reason for the lower median observed in simulations is the presence of a number of vessel segments with zero or negligible RBC fluxes in the primitive vasculature of our developing retina (figure 1*f*), which were absent in the reported experimental data for *adult* mouse retinal capillaries by [41,42]. This major difference will become the focus of study in the following §2.2.

### 2.2. Strong association between RBC hypoperfusion and vessel regression in developing vasculature of mouse retina

**Figure 2.**
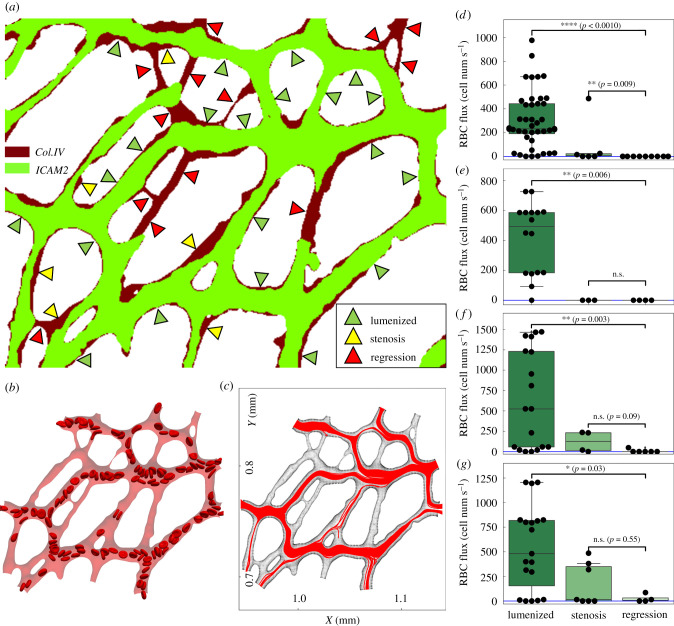
Association between RBC hypoperfusion and vessel regression in the developing retinal network. (*a*) Classification of vessels based on their ICAM2 and Col.IV signals (here demonstrating ROI-1 as in figure 1*a*). All vessels in the ROI are divided into three groups, i.e. lumenized, regression and stenosis. (*b*) A snapshot of the simulation of RBC flow in ROI-1. (*c*) Combined cell trajectories in ROI-1 throughout the simulation (over 0.49 s). (*d*–*g*) Statistical test of time-average RBC flux for the mentioned three groups in ROI-1, ROI-2, ROI-3 and ROI-4, respectively. Two-sided Mann–Whitney *U* test is performed. The sample sizes for the three groups are *n* = 44, 6, 13 in (*d*); *n* = 16, 3, 4 in (*e*); *n* = 18, 4, 6 in (*f*); *n* = 19, 7, 4 in (*g*).

Having provided evidence of the broad range of RBC fluxes in the selected ROIs, we now investigate a potential association between RBC perfusion (referring to the state of vessels being either sufficiently or scarcely perfused by RBCs) and vessel regression within the network. We classify vessels in each ROI into three groups: lumenized, stenosis and regression (figure 2*a*). The ‘lumenized’ group shows positive signals in both Col.IV and ICAM2 throughout the entire vessel segment, indicating stable lumen; the ‘stenosis’ group shows positive signal in Col.IV but partially positive ICAM2 signal that contains at least one location within the vessel segment where less than 50% of the local vessel diameter (as indicated by the local width of Col.IV signal) is covered, featuring focal constriction (an early stage of vessel regression [4]); the ‘regression’ group shows positive signal in Col.IV accompanied by discontinuously positive or overall negative signal in ICAM2 for the vessel segment, indicating partial or complete lumen collapse.

In the four ROIs extracted from the whole plexus (including 63 vessels for ROI-1, 23 vessels for ROI-2, 28 vessels for ROI-3 and 30 vessels for ROI-4), we identified 27 regressed vessels, accounting for 28% out of 97 regressed vessels in total throughout the remodelling region of the plexus (see figure 1*a*). If the regressed vessels and vessels undergoing stenosis within each ROI are counted as remodelling events, the percentages of vessel remodelling within each ROI are 30.4%, 36.7%, 35.7% and 30.2%, respectively. The consistent level of remodelling events within the selected ROIs reflects that the overall remodelling of the primitive vasculature is relatively uniform from region to region.

Further examination of our cellular flow simulations reveals that most poorly RBC-perfused channels (hereafter defined as ‘RBC hypoperfusion’) in the simulation coincide with vessels in the regression group (figure 2*a,b*). To quantify this, we record the trajectories of all RBCs within each ROI throughout the simulation and study their density across the ROIs (figure 2*c* and figure S5 in the electronic supplementary material). In general, a vessel segment with higher density represents good RBC perfusion, whereas those with low density indicate RBC hypoperfusion. Subsequently, the time-average RBC flux within each vessel segment is calculated and assigned to the three groups under study (figure 2*d*–*g*). Our analysis demonstrates that vessel segments in the lumenized group of each ROI have significantly higher RBC fluxes than those in the regression group (*p* < 0.05 for all ROIs, figure 2*d*–*g*). Meanwhile, the difference in RBC flux between the stenosis group and the regression group is not significant (*p* > 0.05) for 3 out of 4 ROIs (except for ROI-1 due to a single outlier, figure 2*d*–*g*). Furthermore, there is no significant difference in the distribution of RBC fluxes between the lumenized groups of these ROIs, despite different maximum values recorded depending on the relative location of the ROI to the artery and the vein (see figure S6a in the electronic supplementary material). These results support a strong association between RBC hypoperfusion and vessel regression within the remodelling plexus.

### 2.3. *In vivo* validation of the effect of RBC perfusion on vascular remodelling in zebrafish caudal vein plexus

**Figure 3.**
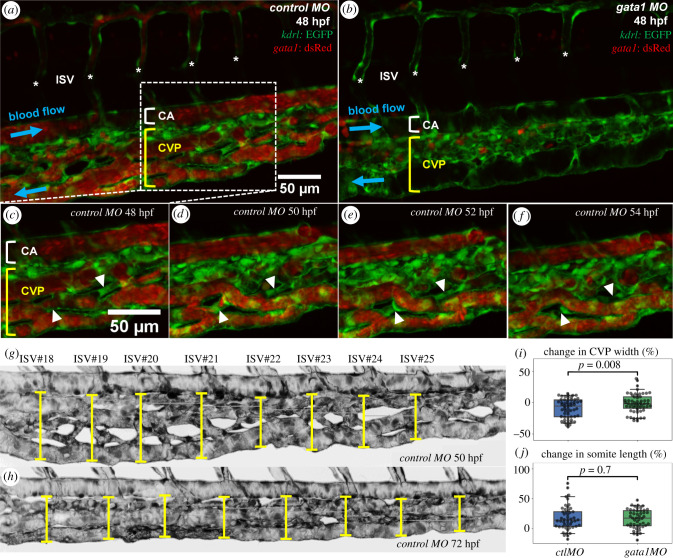
Time-lapse imaging of RBC perfusion and vascular remodelling in zebrafish caudal vein plexus. (*a*,*b*) Two exemplar caudal vein plexuses (CVPs, indicated by a square bracket in yellow) from a 48 hpf ctl MO embryo (with RBC perfusion) and a 48 hpf gata1 MO embryo (*Tg(GATA-1:eGFP)*, without RBC perfusion). The intersegmental vessels (ISVs) are marked with asterisks, and the caudal artery (CA) is indicated by square bracket in white. The RBC precursors in (*b*) are located outside the vasculature and not circulating within the blood stream. (*c*–*f*) Time sequence showing vessel regression events in a region of interest extracted from the ctl MO embryo in (*a*), where two vessel segments marked by white triangles are pruned over time (*t* = 48 hpf, 50 hpf, 52 hpf, 54 hpf). (*g*,*h*) Exemplar measurements of CVP widths (indicated by capped lines) at *t* = 50 hpf and *t* = 72 hpf along the anterior–posterior axis of a ctl MO embryo (Z-projection image) at positions given by eight consecutive ISVs (ISV 18–ISV 25). The lengths of corresponding somites (seven counted here for each fish) are estimated by the inter-ISV distances. (*i*) Normalized CVP width change and (*j*) normalized somite length change at *t* = 72 hpf against *t* = 50 hpf (relative change in percentages), calculated from measurements of the ctl MO group and the gata1 MO group (each containing 7 embryos). The statistical analysis in (*i*,*j*) is performed using Welch’s *T* test, with *n* = 56 in (*i*) and *n* = 49 in (*j*).

To provide experimental confirmation of the association between RBC hypoperfusion and vessel regression predicted by our computational model, we turned to a zebrafish model of vascular development, where simultaneous live imaging of vessel remodelling and RBC dynamics is possible. We chose the caudal vein plexus (CVP) for observation 48–72 h post fertilization (hpf), a period during which gradual remodelling of the plexus down to a single, well-defined vascular tube begins [45]. Comparison was made between control (ctl) morpholino oligomer (MO) fish with normal RBC perfusion (figure 3*a*) and gata1 MO fish not carrying RBCs in the bloodstream (figure 3*b*, see §5.1.2 for experimental details). Our time-lapse imaging of the CVP in ctl MO fish captures heterogeneous RBC perfusion (figure 3*c*) leading to multiple findings of intermittent and complete RBC depletion in vessel segments (figure 3*d*), followed by vessel stenosis (figure 3*e*) and eventual regression (figure 3*f*). These *in vivo* findings therefore confirm our computational predictions.

Next, we investigated how RBC deletion in gata1 MO fish impacts CVP remodelling at a network level. We measured CVP widths at standardized locations along the anterior–posterior fish axis given by the positions of eight consecutive intersegmental vessels (ISVs), for both the ctl MO and gata1 MO groups (seven fishes in each group) at 50 hpf and 72 hpf (see figure 3*g*,*h* for an example). During this period of time, substantial remodelling leading to narrowing of the plexus is observed in the wild type (agreeing with [45]). Furthermore, significantly larger reduction of CVP width in the ctl MO group is found in comparison to the gata1 MO group, accounting for 9% and 1% of relative width change (mean reductions 7.64 µm and 2.01 µm), respectively (*p* < 0.01, figure 3*i*). To exclude that differences in fish growth may confound these results, the longitudinal extension of the somites is also examined, for which no significant difference is found between the ctl MO and gata1 MO groups (*p* > 0.05, figure 3*j*). Taken together, this several-fold difference in CVP width change implies that the presence of RBCs is necessary for normal CVP remodelling 50–72 hpf, and therefore the heterogeneity in RBC perfusion described in figure 3*c*–*f* does play a role in orchestrating network-level remodelling.

### 2.4. RBC depletion in the network not predictive by vessel diameter

The absence of RBCs in some vessel segments in both our simulations and experiments poses a crucial question about cellular flow in developmental vascular network: what is the governing mechanism that determines which vessels to be perfused with cells and which to be devoid of? It is tempting to speculate that the pattern of vessel RBC hypoperfusion is merely a size-exclusion effect; namely, certain vessel segments are simply too narrow to allow cells to pass through. However, the vessel diameters encountered in the present simulations (about 2–20 µm, see figure 1*c*,*e*) are unlikely to be the dominant factor affecting RBC transit, since the high deformability of RBCs under physiological conditions enables them to pass through exceedingly small passages as narrow as 1–2 µm [46–48]. Indeed, the material model we adopt for the RBC membrane in simulations (see benchmark tests in figures S3–S4 and §S1.3 of the electronic supplementary material) allows the cell to adopt highly elongated shapes to pass through nearly all narrow capillaries that they enter (with two occasions of cell occlusion due to negligible flow in the vessel), whereas some larger vessels are devoid of RBCs (see the electronic supplementary material, movies S1–S4).

To further quantify the pattern of RBC perfusion in the network, we define RBC ‘depletion’ and ‘enrichment’ by examining the sign of the term $\Delta Q^{*}=Q_{\mathrm{rbc}}^{*}-Q_{\mathrm{blood}}^{*}$ (see definitions in §5.3.1) for child branches at diverging bifurcations, which evaluates the fractional RBC flux a child branch receives from its parent $Q_{\mathrm{rbc}}^{*}$ relative to its counterpart of fractional blood flow $Q_{\mathrm{blood}}^{*}$. If ΔQ* is negative for a branch, it is regarded as ‘RBC-depleted’ as disproportionately fewer RBCs are allocated to it than the proportion of blood flow it receives; otherwise the branch is defined as ‘RBC-enriched’.

**Figure 4.**
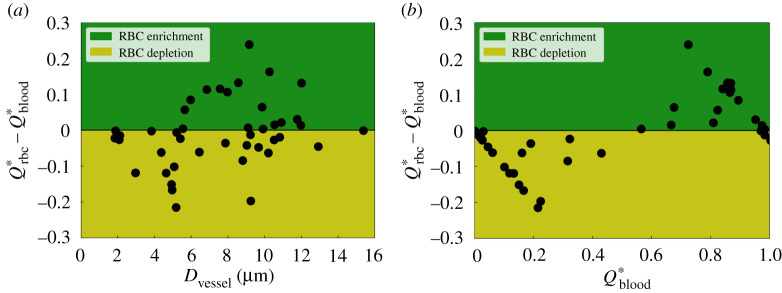
Quantification of RBC hypoperfusion in the developing retinal network. $Q_{\mathrm{rbc}}^{*}$ and $Q_{\mathrm{blood}}^{*}$ represent the normalized RBC flux and normalized blood flow in a given child vessel (with diameter *D*_vessel_) relative to those in its parent vessel, respectively. The variable $\Delta Q^{*}=Q_{\mathrm{rbc}}^{*}-Q_{\mathrm{blood}}^{*}$ serves as a disproportionality index of flow-mediated RBC partitioning, based on the sign of which the vessels are classified as ‘RBC-depletion’ (negative ΔQ*, yellow patch) and ‘RBC-enrichment’ (positive ΔQ*, green patch). The disproportionality indices for all investigated vessel segments are sorted against (*a*) vessel diameter *D*_vessel_ and (*b*) normalized blood flow $Q_{\mathrm{blood}}^{*}$. The analysed vessel segments in this plot are extracted from ROI-2, ROI-3 and ROI-4 (figure 1).

By inspecting ΔQ* against vessel diameter *D*_vessel_ for all valid child branches extracted from ROI-2, ROI-3 and ROI-4 (44 vessels in total, electronic supplementary material, figure S5), we find that RBC depletion occurs throughout the whole range of vessel sizes investigated (*D*_vessel_ ∈ [2, 16] µm, figure 4*a*). Notably, for one child branch with *D*_vessel_ ≈ 9 µm (larger than the physiological RBC diameter), ΔQ* equals −0.2, indicating a 20% reduction of RBC transit relative to the blood flow in it. Based on these findings, we conclude that RBC depletion is not a size-exclusion effect. Meanwhile, it is found that RBC enrichment happens only in medium/large vessels (*D*_vessel_ > 5 µm). Within the intermediate diameter range *D*_vessel_ ∈ [5, 12] µm, the vessels have nearly equal chances of being enriched or depleted.

### 2.5. Plasma skimming as a mechanism for RBC depletion in developing vascular network

Having demonstrated that size-exclusion effect is not sufficient to explain the RBC depletion observed in §2.2, we now turn our attention to potential haemodynamic mechanisms. First, we inspect the RBC fluxes in the vessels against their blood flow rates, and find the two variables nearly monotonically correlated (see figure S6b in the electronic supplementary material). Furthermore, we observe that characterizing $\Delta Q^{*}=Q_{\mathrm{rbc}}^{*}-Q_{\mathrm{blood}}^{*}$ against the haemodynamic indicator $Q_{\mathrm{blood}}^{*}$ satisfactorily separates the RBC-depletion zone from the RBC-enrichment zone (figure 4*b*). This finding is in line with the plasma skimming effect described by empirical models such as the widely employed phase-separation model (PSM, see introduction in §5.3.2), first proposed by Pries and co-workers [49,50].

**Figure 5.**
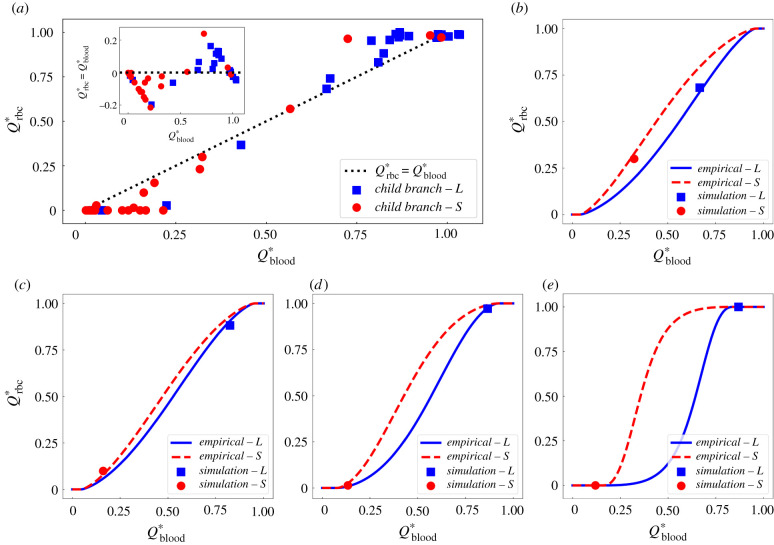
Comparison of simulation data with empirical predictions by the phase separation model [51]. (*a*) Simulation data of fractional RBC flux $Q_{\mathrm{rbc}}^{*}$ against fractional blood flow $Q_{\mathrm{blood}}^{*}$ in the relatively larger child branch ‘L’ (blue squares) and smaller child branch ‘S’ (red circles) from all investigated bifurcations. The inset shows similar results as in figure 4*b* (characterizing the disproportionality index $\Delta Q^{*}=Q_{\mathrm{rbc}}^{*}-Q_{\mathrm{blood}}^{*}$ against $Q_{\mathrm{blood}}^{*}$), but with additional information of relative vessel size for child branches in each bifurcation. The black dotted line represents a linear hypothesis for $Q_{\mathrm{rbc}}^{*}$ and $Q_{\mathrm{blood}}^{*}$ in the absence of plasma skimming. (*b*–*e*) Four exemplar bifurcations in which the simulation data (squares and circles) agree well with empirical predictions (solid lines) for both the ‘L’ and ‘S’ child branches.

To qualitatively assess the agreement between our data and the PSM, we plot the fractional RBC fluxes $Q_{\mathrm{rbc}}^{*}$ in individual child branches (44 studied here) of any divergent bifurcation against their fractional blood flow $Q_{\mathrm{blood}}^{*}$ (figure 5*a*). The distribution is indeed reminiscent of the sigmoidal relationship predicted by the PSM, where $Q_{\mathrm{rbc}}^{*}>Q_{\mathrm{blood}}^{*}$ for $Q_{\mathrm{blood}}^{*}>0.5$ up to a threshold where $Q_{\mathrm{rbc}}^{*}=1$ (and the opposite for $Q_{\mathrm{blood}}^{*}<0.5$ down to $Q_{\mathrm{rbc}}^{*}=0$). Furthermore, albeit with occasional exceptions, the child branch relatively smaller in size (red circles) within a bifurcation tends to receive lower blood flow and consequently fewer RBCs, whereas the larger child branch (blue squares) is more likely to have higher blood flow and attract more RBCs (inset of figure 5*a*). Note that ‘smaller’ or ‘larger’ here is a relative notation between the two child branches within a bifurcation, instead of a measure of the absolute vessel size.

Furthermore, we compare our simulation data at each bifurcation with empirical predictions given by the PSM (equation (5.1) and electronic supplementary material, equations (S1)–(S3)). We observe less than 5% errors for 18 out of 22 bifurcations (see figures S7–S8 for individual comparison and table S4 for complete error evaluation in §S2 of the electronic supplementary material). Quantitatively similar results are obtained if the potential effect of absolute cell volume difference between the RBC model and realistic mouse RBC is also accounted for (see formulation in equations (S4)–(S6), and complete error evaluation against simulation data in table S5 of the electronic supplementary material). Figure 5*b*–*e* demonstrates four cases where good agreement is shown. In the first bifurcation, both child branches have considerable proportions of blood flow (roughly 30% and 70%, respectively) and are well perfused by RBCs, with the fractional RBC fluxes matching the PSM predictions (figure 5*b*). In the second bifurcation, most RBCs ($Q_{\mathrm{rbc}}^{*}\approx 90\text{\%}$) enter the relatively larger child branch as it receives more than 80% of the blood flow from the parent branch (figure 5*c*). In the third and fourth bifurcations (figure 5*d*,*e*), the smaller child branch is nearly devoid of cells as the relatively larger branch attracts almost all RBCs from the feeding vessel owing to its predominantly higher proportion of blood flow ($Q_{\mathrm{blood}}^{*}>85\text{\%}$). Larger than 5% deviations from the PSM are observed in the remaining 4 of the 22 bifurcations studied.

**Figure 6.**
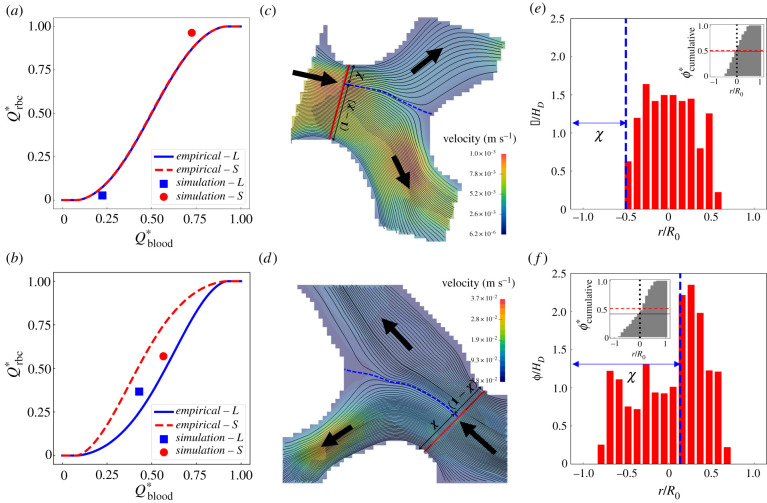
Occasional deviation of simulation data from the empirical model [51] due to the asymmetry of haematocrit profile in the parent branch. (*a*,*b*) Two exemplar divergent bifurcations for which the simulation data (square/circle symbols) deviate from PSM predictions (solid lines). (*c*,*d*) Visualization of the flow streamlines separated into the child branches on the mid-plane of the bifurcation (extracted from the 3D simulation). The blue dashed line indicates the location of the separation surface. (*e*,*f*) Cross-sectional haematocrit profile in the parent branch, at a position marked by the red solid line in (*c*,*d*). The blue dashed line corresponds to the separation surface of flow streamlines as in (*c*,*d*). The insets of (*e*,*f*) show the cumulative haematocrit distribution corresponding to the haematocrit profile.

Shown in figure 6*a*,*b* are two cases where the simulated RBC fluxes in the bifurcation deviate from the empirically predicted values. Interestingly, the RBC flux contrast between the two child branches is slightly underestimated by the PSM in the first case (figure 6*a*) whereas it is substantially overestimated in the second case (figure 6*b*). To investigate these disagreements, we examine the flow streamlines in the mid-plane of the bifurcation and calculate the relative distance *χ* of the stagnation streamline separating the flow entering the left branch from that entering the right branch (figure 6*c*,*d*). Clearly, the higher-flow branch (child branch with larger $Q_{\mathrm{blood}}^{*}$) receives proportionally more streamlines from the parent vessel and should therefore receive more RBCs accordingly provided that the cells are axisymmetrically distributed in the parent branch (as assumed by the empirical PSM).

Next, we investigate whether the assumption of haematocrit axisymmetry by the PSM holds in our simulations. To this end, we calculate the cross-sectional distribution of RBCs in the parent branch. We find clearly asymmetric distributions, especially for case two (figure 6*e*,*f*). In both cases, the haematocrit profile is skewed towards the right-hand side of the bifurcation, with a cumulative haematocrit to the left-hand side of the vessel centreline either slightly or substantially smaller than 0.5 (see insets of figure 6*e*,*f*). Such haematocrit asymmetry makes the downstream child branch on the right-hand side inherently advantageous for RBC intake (hereafter referred to as haematocrit-favoured branch). In case one, the higher-flow branch coincides with the haematocrit-favoured branch, resulting in enhanced RBC flux difference between the two child branches; whereas in case two, the higher-flow branch differs from the haematocrit-favoured branch, thus attenuating the RBC flux difference. A full description of the mechanism leading to this skewness, which originates from the interplay between the complex geometry and the emerging RBC behaviour in the microvasculature, is out of the scope of the present study and will be explored in future work.

## 3. Discussion

Earlier studies have extensively explored the effect of blood flow on cardiovascular development and it is well accepted that haemodynamic cues are essential for vascular development [7,11,12]. Previous work by our groups further established the pivotal role of regional WSS differences, which were found to locally modulate the polarized migration of ECs into high-shear vessel segments and cause the regression of adjacent segments experiencing low shear [4,14,18]. However, because the quantification of WSS in these studies relied on mathematical flow models assuming simplified blood rheology, the question of how the presence of RBCs and their profound impact on microvascular haemodynamics affect vascular patterning has not been addressed. Recent simulations of cellular blood flow in single microvessels have shown that RBCs can non-trivially modify both the mean and oscillatory components of local WSS [23,25], thereby suggesting a salient impact of RBCs on the mechanotransduction of fluid forces during angiogenesis. Based on these findings, we hypothesize that RBCs in the developing mouse retina play an active role in the course of vascular patterning towards a functional network via altering WSS spatially and temporally.

In the current study, we simulated RBC dynamics in the vascular plexus of a wild-type mouse retina at P5 (postnatal day 5). The computed RBC velocities are in good agreement with *in vivo* measurements made in adult mouse retinas [41,42]. However, differences arise in terms of RBC fluxes (more heterogeneous distribution with lower median value, figure 1*f*). We attribute this discrepancy to structural differences between the developmental stage simulated and the adult stage considered for validation. We hypothesize that as the primitive network remodels, the simulated RBC fluxes will become closer to the *in vivo* measurements from adult mice. Future advances in live imaging of the mouse retina model will contribute to elucidating this process.

There are other sources of uncertainty in the current simulations remaining to be quantified. First, the discharge haematocrit at the inlets of all ROIs was fixed at 20% for consistency. This value is in line with the average microvascular haematocrit level reported in the literature, i.e. 23±14% by [52], but may not necessarily reflect the haematocrit levels of individual mice studied in [41,42] and used for validation in our study. Second, the flow in realistic microvessels with smaller lumen size than the RBC diameter is typically impeded due to the close contact of RBCs with the endothelial surface layer (ESL, consisting of glycocalyx and/or cilia), whereas in our present *in silico* model such microscopic structures on the vessel wall are not considered. Prior studies in glass tubes where the ESL was absent demonstrated a twofold decrease in flow resistance when compared with *in vivo* conditions [53,54].

Our simulations of cellular blood flow reveal a previously unreported high-level heterogeneity in RBC perfusion throughout the developing vascular network, where a number of plasma vessels exist with rare RBC transit over time, and a strong association between RBC hypoperfusion and vessel regression. From a mechanistic point of view, the effective viscosity in neighbouring RBC-depleted and RBC-enriched branches can differ substantially. Such disparity in viscosity will enhance the regional WSS difference between neighbouring branches, which can in turn promote the pruning of the vessel segment depleted of RBCs according to our previously reported mechanisms [4].

We provide further experimental confirmation of the above findings in a developmental zebrafish model, which is amenable to live imaging. In agreement with Lucitti *et al.* [7], we show that the presence of RBCs is necessary for effective remodelling at a whole plexus level. Furthermore, we extend the conceptual model by demonstrating that intermittent and complete RBC depletion (also observed recently in a model of inflammatory corneal angiogenesis [55]) selects vessels for pruning, which are likely to experience low shear and become unfavoured during vascular remodelling. Apart from their impact on the WSS differences, Xiong *et al.* [56] recently reported that the signalling role of RBCs also influences vessel remodelling as erythrocyte-specific sphingosine-1-phosphate was found crucial for vascular stabilization and maturation during embryonic development. Understanding the interplay between haemodynamic forces and secreted factors derived from RBCs, however, is beyond the scope of the present study.

To fully understand the perfusion of RBCs within the developing mouse retina, we asked the question: what is the mechanism behind RBC depletion/enrichment in the primitive network? Quantification of RBC perfusion against vessel diameter and blood flow in individual bifurcations rules out vessel-size exclusion as the primary factor. Instead, the empirical model by Pries *et al.* [49,51] based on the plasma skimming effect explains the uneven partitioning of RBC fluxes satisfactorily in 18 out of 22 cases, therefore implying that the distribution of RBCs within the developing network is flow-mediated rather than geometry-dominant.

For the four cases where predictions of the empirical model do not satisfactorily match the simulation data, we find considerable haematocrit asymmetry in the cross-sections of the feeding branches, which is against the central assumption of axisymmetric haematocrit profile by the model [49,51]. This makes accurate prediction of the RBC perfusion in a given vascular network challenging without certain knowledge of cross-sectional cell distributions. Our observation of haematocrit-favoured and haematocrit-unfavoured vessel branches at microvascular bifurcations is in line with recent *in vitro* findings of inversion of the classic haematocrit partitioning, which otherwise always favours the higher-flow branch [57–60]. Similar reverse partitioning was reported by simulations of cellular blood flow in microvascular networks with vessel diameters designed following Horton’s Law [30]. Our group recently identified reduced interbifurcation distance and complex branching topology as sources of haematocrit bias in the context of tumour blood flow, where a role was proposed for the resulting abnormal partitioning in establishing tumour tissue hypoxia [61].

The asymmetry not only applies to the haematocrit profile but also the velocity profile. For a majority of microcirculatory models, the Poiseuille Law has been employed to simplify the haemodynamics and reduce computational cost, manifested by parabolic velocity profiles featuring a peak velocity at the centreline of microvessels. Our results counter such a simplification, as the velocity profile in a capillary vessel can significantly deviate from a parabola and become skewed over time in the presence of travelling RBCs (figure S9 in §S3 of the electronic supplementary material). Similar deviation of velocity profiles has also been confirmed by imaging data from living mouse retina, where a 39% error in flow estimation was reported if assuming a parabolic profile [41].

Finally, the implications of the uncovered association between RBC hypoperfusion and vessel regression are not limited to developmental vascular remodelling. Diseases such as diabetes mellitus and hypertension lead to changes in RBC deformability [62–64] and these have been associated with vascular complications such as diabetic retinopathy or nephropathy in several clinical studies [35–38]. However, the magnitude of the changes in RBC deformability reported in the literature (ranging between 10% and 50% [38,65,66] to several folds [67,68]) would not support a model where such biomechanical changes alone are sufficient to cause vessel occlusion since previous studies showed that order of magnitude changes in shear modulus are required to impede RBC transit through narrow passages [46]. By contrast, our findings support a new concept where changes in the mechanical properties of the RBC membrane leading to abnormal haematocrit partitioning at bifurcations (via altered radial distributions of RBCs [69–71]) would reintroduce, in adult networks, the differences in WSS driving developmental vascular remodelling. Given the cross-species corroboration of important roles of WSS and RBC dynamics in vascular remodelling through different vertebrate model systems, future work should investigate the relevance of the findings to human physiology and whether the predicted WSS differences are sufficient to trigger pathological vascular remodelling.

## 4. Concluding remarks

In summary, our study reports a new mechanism for enhancement of the WSS differences driving vascular remodelling during development. These enhanced differences arise due to the highly heterogeneous distribution of RBCs within the primitive plexus, which is primarily governed by the plasma skimming effect. Additionally, we speculate that vascular remodelling driven by the principle of removing RBC-poor vessels (which are inefficient in transporting oxygen) from the primitive vasculature formed in the course of angiogenesis will ultimately lead to a network layout that avoids portions of the tissue being vascularized but poorly oxygenated. This RBC-driven process, which is highly dynamical and emerging in nature, can importantly contribute to the optimal patterning of vascular networks during development. Conversely, it provides a vascular remodelling mechanism capable of linking changes in RBC deformability reported in diseases such as diabetes mellitus or hypertension and the associated microangiopathic complications, which reintroduces the WSS differences in adult microvascular networks akin to those in developmental stage due to abnormal haematocrit distribution arising from locally impeded RBC perfusion. Beyond these findings, our study also has important implications for the mathematical modelling of microvascular haemodynamics in general. In a network of microvessels, multiple effects inexplicable by continuum flow models (albeit widely employed for modelling blood flow in complex vascular networks by existing studies) occur owing to the particulate nature of blood, i.e. essentially a suspension of RBCs. Conventional assumptions such as Poiseuille Law, velocity-/haematocrit-profile symmetry, spatial-/time-average accuracy are all subject to scrutiny when quantifying flow variables such as effective viscosity and WSS in the microcirculation, especially for vessels with a lumen smaller than the undeformed size of an RBC (i.e. roughly 6–8 µm).

## 5. Material and methods

### 5.1. Mouse and zebrafish experiments

#### 5.1.1. Preparation of mouse retina for binary mask acquisition

The mouse strain used in the present study was C57/BL6J. Mice were maintained at the Max Delbrück Center for Molecular Medicine under standard husbandry conditions. Animal procedures were performed in accordance with the animal license X9005/15. Mouse eyes were collected at P5 and fixed with 4% PFA in PBS for 1 h at 4°C, and retinas were then dissected in PBS. Blocking/permeabilization was performed using Claudio’s Blocking Buffer (CBB) [72], consisting of 1% FBS (Gibco), 3% BSA (Sigma-Aldrich), 0.5% Triton X-100 (Sigma-Aldrich), 0.01% sodium deoxycholate (Sigma-Aldrich), and 0.02% sodium azide (Sigma-Aldrich) in PBS at pH 7.4 for 2 h with rocking at 4°C. Primary antibodies were incubated at the desired concentration in 1 : 1 CBB/PBS with rocking at 4°C overnight and secondary antibodies were incubated at the desired concentration in 1 : 1 CBB/PBS for 2 h at room temperature. Retinas were mounted on slides using Vectashield mounting medium (H-1000; Vector Labs).

The following primary and secondary antibodies were used *in vivo*: collagen IV (ref. 2150-1470, rabbit; 1 : 400; AbD Serotec) and ICAM2 (ref. 553326, rat; 1 : 200; BD Biosciences), anti-Rat Alexa 488 (ref. A21208, donkey 1 : 400, Invitrogen), anti-Rabbit Alexa 568 (ref. A10042, donkey 1 : 400, Invitrogen). Complete high-resolution three-dimensional rendering of whole mount retinas was conducted using a LSM 780 inverted microscope (Zeiss) equipped with a Plan-Apochromat 63 × /1.4 NA DIC objective. Images were taken at room temperature using Zen 2.3 software (Zeiss). Tiled scans of whole retinas were analysed with ImageJ to generate binary masks of ICAM2 and Collagen IV.

#### 5.1.2. Morpholino oligomers, zebrafish husbandry and imaging

Zebrafish (*Danio rerio*) were raised and staged as previously described in [73]. For growing and breeding of transgenic lines, we complied with regulations of the animal ethics committees at the Max Delbrück Center for Molecular Medicine, Berlin [74]. Morpholino oligomer (MO) against *gata1* Morpholino (gata1 MO) as described in [75] (sequence 5’-CTGCAAGTGTAGTATTGAAGATGTC-3’) was injected at 8 ng/embryo following [76]. A control MO (ctl MO) served the standard control Morpholino with the sequence 5’-CCTCTTACCTCAGTTACAATTTATA-3’ targeting a human beta-globin intron mutation. The control was injected at similar amount of 8 ng/embryo.

Embryos were anaesthetized in 0.014% tricaine (Tricaine Pharmaq 1000mg/g, PHARMAQ Limited), mounted in plastic petri dishes (94 × 16 mm, Sarstedt ref. no. 82.1473) containing 0.014% tricaine, and bathed in E3 media containing 0.007 (0.5×) tricaine and 0.003% PTU. Imaging was performed on an upright 3i spinning-disc confocal microscope using Zeiss Plan-Apochromat 20×/1.0 NA water-dipping objectives. Screening of embryos was performed using a Leica M205 FA stereomicroscope with filter set ET GFP M205FA/M165FC.

### 5.2. Numerical simulation

#### 5.2.1. Network characterization and region of interest selection

Given the small size of a single RBC relative to the dimension of the whole-plexus vasculature, direct simulation of cellular blood flow in the entire network would be prohibitively expensive if the grid resolution required to resolve the RBC is applied to the flow domain. Therefore, it is necessary to simulate RBC flow in a reduced number of ROIs for investigation of its effect on vessel remodelling.

There are mainly four criteria for the selection of ROIs from the whole-plexus vasculature without losing generality. First, the selected ROIs should be located within the remodelling region of the plexus (which has been estimated to fall into a range of 0.7 times the radial distance between the optic disc and the farthest vessel in the plexus periphery [40]), thus avoiding the sprouting front where vessel anastomoses occurring due to active EC proliferation could present a similar ICAM2 positive and Col.IV negative signature. Second, the selected ROIs should be representative of different zones within the remodelling area, e.g. close to the feeding arteriole, close to the discharging venule, or relatively central in the capillary bed. Third, each ROI should have plenty of vessel regression or stenosis events for targeted observation of the corresponding RBC dynamics. Fourth, each ROI should be of limited size to allow for sufficient steady-state RBC data (i.e. excluding the transient time steps of the simulation until the RBCs have been fully populated with a stable number of cells count from the simulated ROI) with tractable computational cost.

#### 5.2.2. Whole-plexus simulation

A 3D flow model of the luminal surface (electronic supplementary material, figure S1b) is reconstructed from the Col.IV binary mask (electronic supplementary material, figure S1a) using the open-source software PolNet [44], under the assumption of circular vessel cross-sections. The flow domain is then uniformly discretized into cubic lattice grids with a voxel size of Δ*x*. The lattice resolution is determined as Δ*x* = 0.5 µm (corresponding to a simulation time step length of Δ*t* = 4.17 × 10^−8^ s) for the mouse retina used here such that the flow can be reliably solved in the reconstructed network (containing 39 514 304 voxels in total). We have previously found that accurate flow rates and WSS values can be recovered if the majority of vessels have a diameter of at least 3Δ*x* and 7Δ*x*, respectively [43].

The whole-plexus simulation in the reconstructed network adopts the non-Newtonian Carreau–Yasuda rheology model (NNCY) [77] following our previous approach [43], where blood is modelled as a homogeneous non-Newtonian fluid with shear-thinning behaviour. By imposing a physiological ocular perfusion pressure (OPP) between the artery and veins of our V-A-V type plexus, a steady flow within the vascular network is solved (electronic supplementary material, figure S1c) to provide boundary conditions for subsequent RBC simulations in designated ROIs of the plexus. OPP = 55 mmHg is chosen for the present simulation based on a literature survey and sensitivity analysis conducted in [43]. Electronic supplementary material, table S1, provides key parameters of the whole-plexus simulation. The computational framework based on immersed-boundary lattice-Boltzmann method is detailed in §S1 of the electronic supplementary material.

#### 5.2.3. RBC simulation in network subsets

To create RBC simulations in ROIs of the retinal network where evident vessel regression events are observed (e.g. electronic supplementary material, figure S1d), we first clip the designated ROI from the whole plexus as a geometric subset (electronic supplementary material, figure S1e). For a ROI subset with *N* open boundaries, we set up (*N* − 1) Poiseuille velocity inlets/outlets (where parabolic velocity profiles are imposed with a centreline velocity of $\hat{u}$) and one pressure outlet (where a reference pressure *p*_out_ = 0 is set) (see electronic supplementary material, figure S2, for an illustration of the boundary conditions in ROI-1). The velocity boundary conditions are specified based on the NNCY simulation through integrating local flow rates *Q* at designated vessel cross-sections and subsequently calculating the centreline velocity $\hat{u}$ from *Q* under the assumption of Poiseuille flow, following $\hat{u}=2\bar{u}=8Q/(\pi D_{\mathrm{vessel}}^{2})$. With all the above boundary conditions set up (see electronic supplementary material, table S3), a plasma flow simulation is initiated in the ROI with the fluid viscosity equal to that of plasma *η*_plasma_. Once steady flow is achieved and the velocity field is verified against the NNCY simulation, we populate the ROI with RBCs which are continuously fed at a discharge haematocrit of 20% from all inlets of the subset network (electronic supplementary material, figure S1f). Details of the RBC model can be found in table S2 of the electronic supplementary material.

### 5.3. Data analysis

#### 5.3.1. Vessel selection and quantification of RBC flow

For quantification of RBC perfusion within the selected ROI subsets, we first locate all divergent bifurcations (i.e. composed of one feeding parent branch and two downstream child branches) encountered by the cellular flow in each ROI via exhaustively examining the flow directions in every single vessel segment (ensured identical between the preliminary ROI plasma flow simulation and the whole-plexus simulation) (electronic supplementary material, figure S5a–c). Only vessel branches from these divergent bifurcations are then selected for analysis as representation of the systematic RBC partitioning occurring in the network. In total, 22 divergent bifurcations (excluding bifurcations with excessively short branches or no RBCs involved) and 54 independent vessel segments (excluding 12 repetitive branches) are identified from ROI-2, ROI-3 and ROI-4. To quantify the time-average distribution of RBCs at each divergent bifurcation (electronic supplementary material, figure S5d–f), we introduce two variables following the practice of [30,49]: $Q_{\mathrm{blood}}^{*}$, denoting the proportion of blood flow that a given child branch receives from its parent branch ($0\leq Q_{\mathrm{blood}}^{*}\leq 1$); $Q_{\mathrm{rbc}}^{*}$, denoting the proportion of RBC flux likewise ($0\leq Q_{\mathrm{rbc}}^{*}\leq 1$). If $Q_{\mathrm{rbc}}^{*}>Q_{\mathrm{blood}}^{*}$, the child branch is receiving more RBCs than linear allocation and we define it as an ‘RBC-enriched’ vessel; if $Q_{\mathrm{rbc}}^{*}<Q_{\mathrm{blood}}^{*}$, the child branch is receiving fewer RBCs than the linear hypothesis and we define it as an ‘RBC-depleted’ vessel.

#### 5.3.2. Evaluation of simulation data against the phase separation model

The PSM proposed by Pries and co-workers [49,51] derived a set of empirical formulations based on experimental observation of arteriolar bifurcations in rat mesentery, and established a flow-mediated mechanism to quantitatively describe the RBC fluxes received by child branches of diverging bifurcations within a microvascular network. The PSM correlates the fractional RBC flux *FQ*_*E*_ in a child vessel of a divergent bifurcation with the fractional blood flow *FQ*_*B*_ that it receives:

5.1 $$FQ_{E}=\{\begin{array}{ll} \frac{1}{1+e^{-[A+B\ln((FQ_{B}-X_{0})/(1-(FQ_{B}+X_{0})))]}},\; & X_{0}<FQ_{B}<1-X_{0}\;\\ 0,\; & FQ_{B}\leq X_{0}\;\\ 1,\; & FQ_{B}\geq 1-X_{0},\; \end{array}$$

where *A*, *B* and *X*_0_ are fitting parameters derived via linear regression analysis. Physically, *A* reflects the size difference of the two child vessels, *B* reflects the shape of the haematocrit profile in the parent vessel and *X*_0_ is related to thickness of the cell-free layer near the corresponding vessel wall.

Details of the PSM formulation we have adopted together with the methods and results for evaluating our simulated bifurcations in ROI-2, ROI-3 and ROI-4 against empirical predictions of the PSM are included in §S2 of the electronic supplementary material (including electronic supplementary material, figures S7–S8 and tables S4–S5).

#### 5.3.3. Statistical analysis

Statistical tests are conducted without any removal or modification of the original data. Testing of data normality is performed with the Shapiro–Wilk test and quantile–quantile plots. For normally or approximately normally distributed data, the parametric Welch’s *T* test (without assuming equal variances between two independent samples) is used and the means of data are measured, e.g. figure 3*i*,*j*. For asymmetrically distributed data, the non-parametric Mann–Whitney *U* test is used and the medians of data are measured, e.g. figure 1*d*,*f* and figure 2*d*,*e*,*f*,*g*. All statistical tests are non-paired and two-sided. The difference between groups is considered statistically significant for *p < 0.05 (**p < 0.01, ***p < 0.001, ****p < 0.0001); *p* > 0.05 is considered non-significant (n.s.).

## Data accessibility

Data and information supporting this article are provided in the electronic supplementary material, including figures S1–S9, tables S1–S5, equations S1–S6 and movies S1–S4. The source code for the blood flow simulations is available at https://github.com/hemelb-codes/hemelb.
